# Clinical and epidemiological characteristics of respiratory syncytial virus, SARS-CoV-2 and influenza paediatric viral respiratory infections in southwest Saudi Arabia

**DOI:** 10.1080/07853890.2024.2445791

**Published:** 2024-12-24

**Authors:** Ali Alsuheel Asseri, Saleh M. Al-Qahtani, Ibrahim A. Alzaydani, Ahmed Al-Jarie, Noha Saad Alyazidi, Ali A. Alrmelawi, Alya Musfer Alqahtani, Rahaf S. Alsulayyim, Ameerah K. Alzailaie, Dhay M. Abdullah, Abdelwahid S. Ali

**Affiliations:** a Department of Child Health, King Khalid University, Abha, Saudi Arabia; b Department of Pediatrics, Abha Maternity and Children Hospital, Abha, Saudi Arabia; c Department of Microbiology, Abha Maternity and Children Hospital, Abha, Saudi Arabia; d Department of Pediatric, Khamis Mushate Maternity and Children, Abha, Saudi Arabia; e Health Affairs, Aseer Region, Abha, Saudi Arabia; f King Fahad Armed Hospital, Southern Region, Abha, Saudi Arabia; g Department of Microbiology and Clinical Parasitology, College of Medicine, King Khalid University, Abha, Saudi Arabia

**Keywords:** Respiratory syncytial virus, SARS-CoV-2, influenza, children, epidemiology, clinical manifestation, Saudi Arabia

## Abstract

**Background:**

There is a global consensus that respiratory tract infections are the major causes of morbidity and mortality among children. In this study, we aimed to compare the clinical and epidemiological characteristics of respiratory syncytial virus (RSV), influenza and severe acute respiratory syndrome coronavirus 2 (SARS-CoV-2) infections among children admitted to hospital with acute respiratory infections. We also opted to identify the predictors of paediatric intensive care unit (PICU) admission.

**Methods:**

In this study, a retrospective investigation and analysis of 423 children who were admitted to Abha Maternity and Children Hospital, in the southern region of Saudi Arabia, between January and December 2022 were conducted.

**Results:**

The median age of these children was 16.5 months (Q1–Q3: 6–46.3). It was observed that the infectivity levels of RSV, SARS-CoV-2, influenza A and influenza B infections peaked in early to mid-September, mid-July, May and June, and October, respectively. There was a statistically significant difference in the total WBC counts between RSV and influenza B (*p* = 0.035) and SARS-CoV-2 and influenza B (*p* = 0.013). Moreover, there was a statistically significant difference in the absolute lymphocyte count between influenza A and RSV (*p* = 0.002). The median number of days in hospital was 6 days (Q1–Q3: 4–10). Patients with RSV infection required a significantly longer hospital stay, with a median of 8 days (Q1–Q3: 4–10). The factors associated with the likelihood of PICU admission for all study participants were congenital heart disease (odds ratio (OR) = 2.9, 95% confidence intervals (CI) [1.4–6.1]), RSV (OR = 2.3, 95% CI [1.3–4.1]) and age <6 months (OR = 2.0, 95% CI [1.2–3.4]).

**Conclusions:**

RSV was identified as the most common pathogen causing acute lower respiratory infections among the studied patients. One of the more significant findings to emerge from this study is the seasonal changes in RSV and influenza infections, which mandates further research.

## KEY MESSAGES

1. RSV is the leading cause of lower respiratory infections in children, with a crucial finding of distinct seasonal peaks for RSV and influenza infections during the studied period.
2. The presence of lymphopenia in patients with viral infection, particularly influenza and SARS-CoV-2, could be used as a predictor of disease severity.

## Introduction

There is a global consensus that respiratory tract infections are the major causes of morbidity and mortality among paediatric populations worldwide [1–3]. Human respiratory syncytial virus (hRSV) is regarded as the leading cause of acute and fatal paediatric lower respiratory tract infections among infants and young children in many parts of the world [4–7]. hRSV is an enveloped, negative-sense, single-stranded RNA virus and a member of the Pneumoviridae family, Pneumovirinae subfamily and Orthopneumovirus genus [8]. RSV infections have been documented as major attributes of morbidity and mortality among children in many epidemiological surveys, and as being responsible for major healthcare burdens [9–11] in paediatric medicine. It has been established that the clinical courses of RSV infections range from mild to severe, critical, or fatal due to bronchiolitis and bronchopneumonia [12–14]. Several patient-related factors are incriminated in the severity of RSV infections and hospitalization rates among paediatric patients, including the age of children [15,16], immune status [16,17] and concomitant viral infections [18]. The clinical profiles and epidemiology of RSV among the elderly and high-risk adults have also been presented in some reports [19].

Influenza virus infections are also known to cause severe infections and high hospitalization rates among child populations in many parts of the world, particularly in resource-limited countries [14,20]. It was documented in several previous studies that influenza can cause critical and fatal infections including pneumonia and bronchopneumonia especially among the neonates and the immune-compromised paediatric patients. The seasonality of influenza infections was also confirmed and reported to occur during the cold winters [11,14,15,20]. Application of routine vaccination policy against influenza among schoolchildren was suggested and recognized of tremendous beneficial effects [21]. Recently, severe acute respiratory syndrome coronavirus 2 (SARS-CoV-2) has also been reported to cause different clinical outcomes and epidemiological patterns among children [22]. Although SARS-CoV-2 infections among children were known of asymptomatic nature at the early days of the pandemic and shortly thereafter, later clinical cases of paediatric SARS-CoV-2 were reported from many countries of the world [22]. Severe disease or a post-infectious multisystem hyperinflammatory syndrome in children has been described [22–25]. In Saudi Arabia, the public health impacts of paediatric viral respiratory infections (VRIs) have been studied and published in various reports [23–27]. In this study, we aimed to compare the clinical and epidemiological characteristics of RSV, SARS-CoV-2 and influenza among children admitted to hospital with acute respiratory infections in the southwestern region of Saudi Arabia. We also opted to identify the predictors of paediatric intensive care admission.

## Materials and methods

### Setting, design and study population

This retrospective observational study reviewed all cases of RSV, SARS-CoV-2, influenza A and influenza B infections detected in patients aged 1 month to 12 years who were admitted to the general wards or the paediatric intensive care unit (PICU) of Abha Maternity and Children Hospital (AMCH) with acute lower respiratory infection (ALRI) between January and December 2022. According to the WHO definition [28], ALRI was defined based on the presence of acute respiratory symptoms reported within the last 10 days, including a history or measured fever of ≥38ºC, cough and shortness of breath. Viral infection was confirmed using the reverse-transcriptase polymerase chain reaction test on a nasopharyngeal swab specimen.

### Data collection and criteria for hospitalization

The data collection sheets consisted of three sections: clinical and demographic variables, laboratory and radiographic imaging findings and outcome measures. The clinical and demographic variables included age, gender, history of contact with a sick patient, symptoms, comorbid medical conditions and initial emergency room physical signs. Hypoxemia, considered another inclusion criterion, was present in all patients and defined as peripheral oxygen saturation (SpO_2_) via pulse oximetry [28]. The laboratory examinations, including blood tests, bacterial culture and chest radiographs, were ordered according to the physician’s discretion. The chest radiological findings were evaluated by senior paediatric radiologist, using consolidation, over-inflation and unilateral or bilateral findings as endpoints. The admission criteria were based on the physician’s decision. However, the majority of the reasons for admission included a requirement for supplemental oxygen due to hypoxia, a requirement for hydration due to intolerable oral feeding and/or a poor general condition.

### Statistical analysis

The SPSS software program version 29 (IBM SPSS Statistics for Windows, Armonk, NY, USA: IBM Corp) was used for statistical analysis. Normally distributed variables are presented as the mean and standard deviation, while non-normally distributed variables are presented as the median with the interquartile range (IQR). The Shapiro–Wilk test and histogram data visualization were used to assess the normality of the continuous variables. Counts and percentages are used to represent the categorical variables. Continuous variables are presented as the mean ± SD (standard deviation) and the median with the IQR for normally and non-normally distributed variables, respectively. Statistical analysis of the differences was performed using Pearson’s chi-square (χ^2^) test and Fisher’s exact test for categorical variables, as appropriate. The one-way analysis of variance (ANOVA) test was used to compare the means, followed by Bonferroni’s post hoc test, and for non-normally distributed data, the Kruskal–Wallis *H* test was used. Univariable and multivariable binary logistic regression models were used to identify the factors associated with the need for PICU admission. The results are reported as odds ratios (ORs) and their 95% confidence intervals (CIs). The dependent variable was ‘needing PICU admission’. The analysis included a set of independent variables categorized as dichotomous (yes/no). The independent variables included in the study were age <6 months, RSV infection, congenital heart disease (CHD), chronic lung disease (CLD), central nervous system disease (CNS), airway anomalies, prematurity and SpO_2_ <90%. The reference group for each variable was designated as ‘no’. Significant differences were considered when the *p*-value was <0.05.

## Results

### Clinical and epidemiological characteristics of the study population

**Figure 1.**
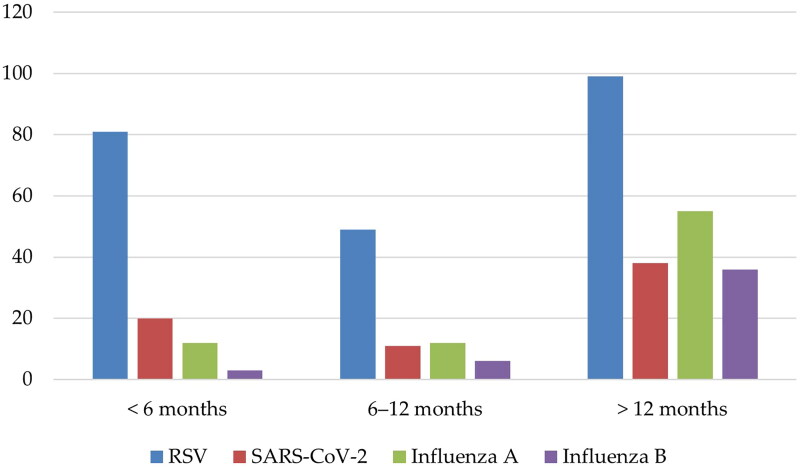
Age distribution of RSV, severe acute respiratory syndrome coronavirus 2 (SARS-CoV-2) and influenza (A and B) infections among the enrolled children according to the age groups <6 months, 6–12 months and >12 months.

**Figure 2.**
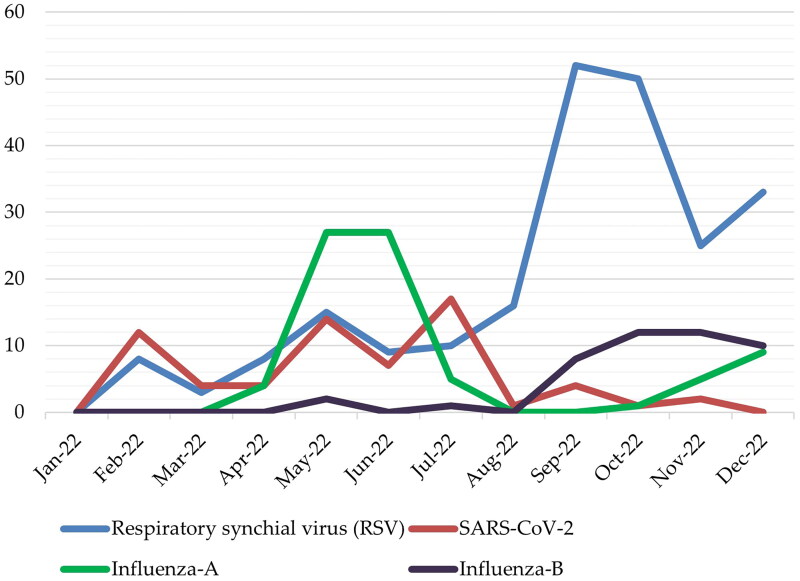
Epidemic curves of RSV, severe acute respiratory syndrome coronavirus 2 (SARS-CoV-2) and influenza (A and B) infections among hospitalized children from 1 January 2022 to 31 December 2022.

Table 1 and Figure 1 show the clinical and epidemiological characteristics of the enrolled patients. This study included 423 children who were enrolled between January and December 2022. The median age of these children was 16.5 months (Q1–Q3: 6–46.3), and they all required hospitalization due to severe ALRI at AMCH, Abha city, southern region of Saudi Arabia. The ages differed significantly between the four studied viral infections (*p* < 0.001); children with RSV were younger (median age, 12 months) in comparison to those with SARS-CoV-2 (median age, 15 months), influenza A (median age, 36 months) and influenza B (median age, 48 months). There had been significant differences noted among the four viral infections in the groups of children of <6 months and >12 months age categories (*p* < 0.001). Of the children <6 months and >12 months, 35.2% had RSV and 80% had influenza B infection, respectively. There was no statistically significant difference between the infections regarding the sex of the patients, contact with a sick person or symptom durations before being hospitalized. The most common symptoms were fever, cough, dyspnoea, rhinorrhoea, feeding problems and wheezing. These symptoms were observed in 96.5%, 90.1%, 84.6%, 83.4%, 78.7% and 65.7% of the total number of patients, respectively. All symptoms differed significantly between the patients (*p* < 0.05), except fever. The prevalence of prematurity differed significantly between the four studied viral infections (*p* < 0.001); children with RSV had a higher prevalence of prematurity (27.8%) in comparison to those with SARS-CoV-2 (21.7%), influenza A (12.7%) and influenza B (2.2%). Children with SARS-CoV-2 were noted to have a greater prevalence of underlying CLD (29%), neurological disorders (27.5%) and airway disorders (15.9%) than children with other infections (*p* < 0.05). A higher proportion of children with influenza A had underlying asthma (34.2%) as compared to those with RSV (19.6%), SARS-CoV-2 (14.5%) and influenza B (15.6%) (*p* = 0.016). The initial oxygen saturation level differed significantly between the four studied viral infections (*p* < 0.001); children with RSV had more severe hypoxia (median SpO_2_, 84%) in comparison to those with SARS-CoV-2 (median SpO_2_, 89%), influenza A (median SpO_2_, 89%) and influenza B (median SpO_2_, 90%). Figure 2 shows the number of cases of each pathogen per month. It was observed that the infectivity levels of RSV, SARS-CoV-2, influenza A and influenza B peaked in early to mid-September, mid-July, May and June, and October, respectively.

**Table 1.** Characteristics of the enrolled patients stratified according to the type of infection.

Variables	All cases *n* = 423	RSV *n* = 230 (54.3%)	SARS-CoV-2 *n* = 69 (16.3%)	Influenza A *n* = 79 (18.7%)	Influenza B *n* = 45 (10.6%)	*p*-Value
Age, median (IQR), months	16.5 (6–46.3)	12 (9–72)	15 (6–42)	36 (5–36)	48 (5–36)	<0.001
<6 months, *n* (%)	116 (27.4)	81 (35.2)	20 (28.9)	12 (15.2)	3 (6.7)	<0.001
6–12 months, *n* (%)	78 (18.4)	49 (21.3)	11 (15.9)	12 (15.2)	6 (13.3)	0.380
>12 months, *n* (%)	228 (53.9)	99 (43.0)	38 (55.1)	55 (69.6)	36 (80.0)	<0.001
Sex, male, *n* (%)	216 (51.1)	118 (51.3)	28 (40.6)	48 (60.8)	22 (48.9)	0.139
History of contact with sick patient, *n* (%)	382 (90.3)	212 (92.2)	62 (89.9)	69 (87.3)	39 (86.7)	0.130
Onset of symptoms to hospitalization (days), median (IQR)	3 (3–4)	3 (3–4)	3 (3–4)	3 (3–4)	4 (3–4)	0.131
Symptoms, *n* (%)						
Fever	408 (96.5)	218 (94.8)	66 (95.7)	79 (100)	45 (100)	0.160
Wheeze	278 (65.7)	164 (27.8)	47 (68.1)	46 (58.2)	21 (46.7)	0.002
Cough	381 (90.1)	219 (95.2)	56 (81.2)	64 (81.0)	42 (93.3)	<0.001
Rhinorrhoea	353 (83.4)	202 (87.8)	58 (84.1)	61 (77.2)	32 (71.1)	0.001
Dyspnoea	358 (84.6)	220 (95.7)	53 (76.8)	54 (68.4)	31 (68.9)	<0.001
Feeding intolerance	333 (78.7)	206 (89.6)	54 (78.3)	53 (67.1)	20 (44.4)	<0.001
Coexisting medical conditions, *n* (%)						
Premature <37 weeks GA	90 (21.3)	64 (27.8)	15 (21.7)	10 (12.7)	1 (2.2)	<0.001
Chronic lung disease	85 (20.1)	37 (16.1)	20 (29.0)	21 (26.6)	7 (15.6)	0.036
Asthma/RAD	89 (21.0)	45 (19.6)	10 (14.5)	27 (34.2)	7(15.6)	0.016
Airways disorders	39 (9.2)	13 (5.7)	11 (15.9)	11 (13.9)	4 (8.9)	0.025
Congenital heart diseases	54 (12.8)	31 (13.5)	12 (17.4)	6 (7.6)	5 (11.1)	0.275
Neurological disorders	70 (16.5)	27 (11.7)	19 (27.5)	17 (21.5)	7 (15.6)	0.009
Trisomy 21/genetic disorders	63 (14.9)	28 (12.2)	12 (17.4)	13 (16.5)	10 (22.2)	0.091
Vital signs at admission						
SpO_2_ (%), median (IQR)	85 (80–90)	84 (79–87)	89 (81–94)	89 (83–93)	90 (85–93)	<0.001
RR (/min), mean ± SD	43.9 ± 15.1	48 ± 15.4	43 ± 11.9	37.7 ± 13.2	33.7 ± 12.7	<0.001
HR (/min), mean ± SD	145.8 ± 4.5	152 ± 21.8	146.2 ± 18.9	137 ± 21.7	130.7 ± 20	<0.001
Body temperature (°C), median (IQR)	38 (37.5–39)	38 (37.4–38.8)	39 (37–39)	39 (38–39)	38 (37–39)	<0.001

Values of *p* < 0.05 are statistically significant.

RSV: respiratory syncytial virus; *n*: number; SARS-CoV-2: severe acute respiratory syndrome coronavirus 2; IQR: interquartile range; GA: gestational age; RAD: reactive airway disease; SpO_2_: saturation of peripheral oxygen; RR: respiratory rate; HR: heart rate.

### Laboratory and radiographic imaging findings

The laboratory and radiographic imaging findings of the studied participants are listed in Table 2. There was a significant difference between the four viral infection groups of children in the mean of the following laboratory parameters: total white blood cell counts (WBC), absolute lymphocyte counts (ALCs) and platelet counts (*p* < 0.05). Based on the Bonferroni adjustment, there was a statistically significant difference in the total WBC counts between RSV and influenza B (*p* = 0.035) and SARS-CoV-2 and influenza B (*p* = 0.013). Moreover, there was a statistically significant difference in the ALCs between influenza A and RSV (*p* = 0.002) and influenza A and SARS-CoV-2 (*p* = 0.018), and in the total platelet counts between influenza A and RSV (*p* < 0.001), influenza B and RSV (*p* < 0.001), influenza A and SARS-CoV-2 (*p* = 0.013) and influenza B and SARS-CoV-2 (*p* = 0.006).

**Table 2.** Laboratory and imaging characteristics of the study population.

Variables	All cases *n* = 423	RSV *n* = 230 (54.4%)	SARS-CoV-2 *n* = 69 (16.3%)	Influenza A *n* = 79 (18.7%)	Influenza B *n* = 45 (10.6%)	*p*-Value
WBC (ref: 4.3–11.0 × 103/μL), mean ± SD	8.9 ± 4.5	9.3 ± 4	9.8 ± 5.8	8.1 ± 4.3	7.2 ± 4.3	0.004
ANC (ref: 1500–8500 cells/µL), mean ± SD	4477 ± 3552	4342 ± 3146	5222 ± 4614	4750 ± 3872	3355 ± 2566	0.069
ALC (ref: 970–3960/μL), mean ± SD	3475 ± 2579	3756 ± 2457	3764 ± 3125	2525 ± 1956	3232 ± 2871	0.002
Haemoglobin (ref: 11.5–15.5 g/dL), mean ± SD	12 ± 1.8	12 ± 1.8	12 ± 1.5	12.3 ± 2	12.1 ± 1.6	0.377
ESR (ref: 0.0–15 mm/h), mean ± SD	36.5 ± 28	34.8 ± 23	39 ± 31	35.8 ± 30	40 ± 38	0.659
Platelets (×1000/mm^3^), mean ± SD	341 ± 148	368 ± 139	369 ± 191	294 ± 116	267 ± 123	<0.001
ALT (ref: 10–35 U/Lb), median (IQR)	20 (31–54)	19 (14–26)	20 (16–28)	22 (16–42)	26 (19–38)	0.009
AST (ref: 10–34 U/L), median (IQR)	40 (15–28)	42 (30–48)	39 (31–57)	46 (36–96)	42 (38–76)	0.003
Blood urea nitrogen (mg/dL), median (IQR)	12.5 (8–20)	12 (6–19)	14 (9–23)	13 (10–21)	12 (10–17)	0.014
Serum creatinine (mg/dL), median (IQR)	0.23 (0.2–0.3)	0.23 (0.2–0.3)	0.2 (0.2–0.3)	0.2 (0.2–0.4)	0.3 (0.2–0.5)	0.262
Positive blood culture, *n* (%)	25 (6.0)	14 (6.1)	4 (5.8)	3 (3.8)	4 (8.9)	0.900
Positive urine culture, *n* (%)	17 (4.0)	9 (4.0)	4 (5.8)	2 (2.5)	2 (4.4)	0.786
Chest radiographic findings, *n* (%)						
Normal	92 (21.7)	14 (6.1)	23 (33.3)	32 (40.5)	23 (51.1)	<0.001
Hyperinflation	261 (61.7)	184 (80)	37 (53.6)	33 (41.8)	7 (15.6)	<0.001
Atelectasis	207 (48.9)	127 (55.2)	37 (53.6)	34 (43.0)	9 (20.0)	0.002
Consolidation	193 (45.6)	105 (45.7)	35 (50.7)	35 (44.3)	18 (40.0)	0.670

Values of *p* < 0.05 are statistically significant.

RSV: respiratory syncytial virus; *n*: number; SARS-CoV-2: severe acute respiratory syndrome coronavirus 2; WBC: white blood count; ref: reference; SD: standard deviation; ANC: absolute neutrophil count; ALC: absolute lymphocyte count; ESR: erythrocyte sedimentation rate; ALT: alanine aminotransferase; IQR: interquartile range; AST: aspartate aminotransferase.

The laboratory findings also showed a significant difference between the four viral infection groups in the following laboratory parameters: alanine aminotransferase (ALT), aspartate aminotransferase (AST) and blood urea nitrogen (*p* < 0.05). The obtained chest radiography (CXR) results indicated statistically significant associations between the radiographic findings and the four viral infection groups regarding the normality of the CXR and the presence of hyperinflation and atelectasis (*p* < 0.05). Out of the total number of children infected with influenza B, 51.1% had a normal CXR, while of those infected with RSV, SARS-CoV-2 and influenza A, 6.1%, 33.3% and 40.5% showed a normal CXR (*p* < 0.001). The most common CXR finding in children infected with RSV was hyperinflation, followed by the presence of atelectasis, both of which were statistically significant (*p* < 0.001 and *p* = 0.002, respectively). The presence of consolidation did not differ between the four groups (*p* = 0.670). When blood and urine cultures were employed, 6% and 4% of the total patient specimens showed positive results, respectively. However, no significant differences in the positive cases among the four groups of patients were observed.

### Treatment and outcome measures

Patients with RSV infection received significantly higher rates of bronchodilator therapy (95.2%) compared with patients with SARS-CoV-2 (62.3%), influenza A (74.7%) and influenza B (64.4%) (*p* < 0.001). During the hospital stays, 94.3% of the patients from all groups were treated with antibiotics (RSV, 90.4%; SARS-CoV-2, 95.7%; influenza A, 100% and influenza B, 100%). The median length of oxygen therapy was 6 days (Q1–Q3: 4–10), and it was significantly different between the four groups of patients (*p* < 0.001). A total number of 115 patients (27.2%) were admitted to the PICU for the initiation of high-flow nasal cannula (HFNC) (RSV, 35.7%; SARS-CoV-2, 20.3%; influenza A, 10.1% and influenza B, 24.4%), which differed significantly between the four groups (*p* < 0.001). Eighteen patients in all groups were noted to require mechanical ventilation due to severe respiratory failure and failure of HFNC, with no significant differences between all the groups of patients. Patients with influenza B infection required a significantly longer PICU stay, with a median of 9 days (Q1–Q3: 2–68) (*p* < 0.001). The median number of days in hospital was 6 days (Q1–Q3: 4–10). Patients with RSV infection required a significantly longer hospital stay, with a median of 7 days (Q1–Q3: 4–10). Out of the 29 patients who went home on oxygen, 18 (62.1%) patients had been on chronic home oxygen prior to the illness. Among the four groups of patients, five patients died: three with RSV and underlying cardiopulmonary comorbidities, one with influenza B infection complicated with seizure disorders and severe ARDS, and one with SARS-CoV-2 on top on underlying trisomy 21 and severe pulmonary hypertension. A summary of the used treatment methods and outcome measures is presented in Table 3.

**Table 3.** Therapies and outcome measures of the enrolled patients.

Variables	All cases (*n* = 423)	RSV *n* = 230 (54.4%)	SARS-CoV-2 *n* = 69 (16.3%)	Influenza A *n* = 79 (18.7%)	Influenza B *n* = 45 (10.6%)	*p*-Value
Nebulizer bronchodilators, *n* (%)	350 (82.7)	219 (95.2)	43 (62.3)	59 (74.7)	29 (64.4)	<0.001
Broad-spectrum antibiotics, *n* (%)	399 (94.3)	208 (90.4)	66 (95.7)	79 (100)	45 (100)	0.001
Length of oxygen therapy (days), median (IQR)	6 (4–9)	6 (4–10)	6 (3–10.3)	5 (3–6)	5 (2–15.5)	<0.001
Intensive care unit admission, *n* (%)	115 (27.2)	82 (35.7)	14 (20.3)	8 (10.1)	11 (24.4)	<0.001
HFNC, *n* (%)	115 (27.2)	82 (35.7)	14 (20.3)	8 (10.1)	11 (24.4)	<0.001
Length of HFNC use (days), median (IQR)	4 (3–6)	4 (2.3–5)	4 (3–5)	4.5 (3.8–9)	8.5 (4–47.5)	0.002
Use of mechanical ventilation, *n* (%)	18 (4.3)	12 (5.2)	1 (1.4)	2 (2.5)	3 (6.7)	0.426
Length of PICU stay (days), median (IQR)	5 (3–8)	5 (3–8)	5 (3–7)	7.5 (3.8–11)	9 (2–68)	<0.001
Length of hospital stay (days), median (IQR)	6 (4–10)	7 (4–10)	5 (3–10)	5 (3–7)	4 (3–11.5)	<0.001
Outcome of hospitalization, *n* (%)						
Discharged	416 (98.3)	225 (97.8)	65 (94.2)	79 (100)	44 (97.8)	0.621
Discharged on home oxygen	29 (6.9)	17 (7.4)	6 (8.7)	4 (5.1)	2 (4.4)	0.698
Died	5 (1.2)	3 (1.3)	1 (1.4)	0 (100)	1 (2.2)	ــــ

Values of *p* < 0.05 are statistically significant.

RSV: respiratory syncytial virus; *n*: number; SARS-CoV-2: severe acute respiratory syndrome coronavirus 2; IQR: interquartile range; HFNC: high-flow nasal cannula; PICU: paediatric intensive care unit.

### Factors associated with intensive care unit admission

Table 4 shows the results of the univariable and multivariable logistic regressions of the variables that predicted PICU admission, along with the unadjusted and adjusted ORs and corresponding significance levels. These factors included age <6 months, RSV infection, CHD, CLD, CNS morbidity, airway anomalies, prematurity and SpO_2_ <90%. All of these factors were found to be associated with PICU admission (all *p*-values were <0.05). The logistic regression model was statistically significant (χ^2^ (8) = 57.181, *p* < 0.001). The model explained 18.7% (Nagelkerke *R*^2^) of the variance in PICU admission and correctly classified 74.8% of the cases. The factors associated with the likelihood of PICU admission for all study participants, in the order of the highest to the lowest ORs, were CHD (OR = 2.9, 95% CI [1.4–6.1]), RSV (OR = 2.3, 95% CI [1.3–4.1]) and age <6 months (OR = 2.0, 95% CI [1.2–3.4]).

**Table 4.** Logistic regression of unadjusted and adjusted odds ratios for patients who required paediatric intensive care unit admission.

Variables		Univariable			Multivariable		
	Comparison	OR	95%CI	*p*-Value	aOR	95%CI	*p*-Value
Age <6 months	Yes vs. no	2.0	1.2–3.0	0.006	2.0	1.2–3.4	0.008
RSV	Yes vs. no	2.7	1.7–4.3	<0.001	2.3	1.3–4.1	0.006
CHD	Yes vs. no	4.4	2.4–8.0	<0.001	2.9	1.4–6.1	0.006
CLD	Yes vs. no	2.3	1.4–3.8	<0.001	1.2	0.6–2.7	0.601
CNS	Yes vs. no	2.3	1.3–3.9	<0.002	1.9	0.8–4.3	0.119
Airway anomalies	Yes vs. no	2.2	1.1–4.3	0.024	0.9	0.3–2.5	0.863
Prematurity	Yes vs. no	1.8	1.1–2.9	0.019	1.1	0.6–1.8	0.992
SpO_2_ < 90%	Yes vs. no	3.9	2.1–7.2	<0.001	1.9	0.9–4.1	0.073

The Hosmer–Lemeshow test showed an acceptable model fit (X^2^ = 3.394, *p* = 0.846). The multivariable logistic regression model included all other variables in the table.

Values of *p* < 0.05 are statistically significant.

OR: odds ratio; aOR: adjusted odd ratio; CI: confidence interval; RSV: respiratory syncytial virus; CHD: congenital heart disease; CLD: chronic lung disease; CNS: central nervous system diseases; SpO_2_: saturation of peripheral oxygen.

## Discussion

VRIs constitute major infectious threats among child populations worldwide. ALRIs are responsible for significant morbidity and mortality in children and represent the primary cause of hospitalization, particularly among children <5 years of age [12,29,30]. Since the beginning of the SARS-CoV-2 pandemic, a notable change in the seasonal distributions of respiratory viral pathogens has been observed [13,31,32]. In this study, we compared the clinical features, epidemiological trends, laboratory findings and chest radiology outcomes of RSV, SARS-CoV-2 and influenza infections in paediatric patients hospitalized with acute LRIs at AMCH in the southwestern region of Saudi Arabia. The results of this study provide important insights into the alterations in the clinical and epidemiological characteristics of four viral respiratory pathogens (RSV, influenza A and B and SARS-CoV-2) at the resurgence of VRIs during the SARS-CoV-2 pandemic.

Our results demonstrated that the most common VRIs reported among the studied children was RSV infection (54.3%), followed by influenza A (18.7%), SARS-CoV-2 (16.3%) and influenza B (10.6%). This was found to be consistent with several previously published reports in different geographical locations around the world [13,33–36]. RSV stood out as being the major pathogen causing ARIs, particularly in patients <6 months. Furthermore, our results are consistent with those of a study conducted in the Eastern Province of Saudi Arabia between January 2015 and February 2022, which found that RSV infection was diagnosed in the majority of hospitalized infants and young children [32]. The results obtained also showed that children hospitalized most commonly with RSV infections were younger in age as compared to those hospitalized with influenza and SARS-CoV-2. These results match those observed in earlier similar studies [13,33].

Another important finding is that RSV infection peaked in early to mid-September, SARS-CoV-2 infection peaked in mid-July, influenza A infection peaked in May and June and influenza B infection peaked in October. This information suggests that the SARS-CoV-2 pandemic has played some roles in the changes in the prevalence and seasonal patterns of paediatric respiratory infections over the last 2 years. Similar previous studies arrived at the same conclusion, finding that SARS-CoV-2 pandemic has undoubtedly contributed to these changes [13,32,35,36]. We assume that the protective measures taken during the pandemic are the major contributing factors in the VRIs’ pattern.

A comprehensive review of the epidemiology of VRIs in Saudi Arabia in the pre-pandemic period showed that RSV infection is the most common from October to March [37]. The change in the seasonal pattern of RSV during the SARS-CoV-2 era noted in this study has also been observed in previous studies [33,35]. Multiple factors may underlie the magnitude of the out-of-season RSV surge. These factors include viral and non-viral aetiologies. The proposed viral hypotheses is that more transmissible or virulent variants of viruses have emerged. Additionally, the non-viral causes include changes in RSV testing, decreased population-level RSV immunity and the altered social networks with the ending of the pandemic restrictions [38,39]. With respect to the proposed viral hypothesis, studies on genomically characterized RSV infections have concluded that the 2022 RSV surge consisted of numerous pre-existing viral variants, which is inconsistent with the emergence of a new, highly transmissible RSV variant as the cause of the surge [38]. Regarding the low RSV immunity, it has been proposed that the interruption of RSV circulation following the pandemic restrictions led to a decrease in population immunity [40]. Additional research confirmed a significant decline in RSV antibodies among infants and childbearing-age women during the SARS-CoV-2 pandemic [41]. Taken together, these data support the notion of immunity debt, which could be a potential explanation for the RSV surge; however, more studies are needed to further explore these findings and propose proper prevention strategies. Some of the issues emerging from these findings specifically relate to the timing of the annual influenza vaccine and the seasonal RSV immunoprophylaxis programme. These preventive measures are usually recommended prior to the winter months; however, the new seasonal trends of RSV and influenza infections mandate further longitudinal data at the national level to clearly recommend the best timing of these preventive measures.

This study revealed that leukopenia, lymphopenia and thrombocytopenia were more frequent in influenza A and B patients than in patients with other infections. All patients had CXRs, and 45.6% had confirmed pneumonia, with 50.7% of them having SARS-CoV-2 infection. Abnormalities in the chest X-rays, such as hyperinflation and atelectasis, were more often present in RSV patients than those with influenza and SARS-CoV-2. Laboratory markers can indicate the severity of the infection and help to predict patient outcomes. Lymphopenia has been reported as a strong predictor of several viral infections [42]. Experimental studies have investigated a possible mechanism for lymphopenia after influenza virus infection, focusing on the thymus as a primary lymphoid organ, and they concluded that direct thymus invasion by the influenza virus and possible other viruses could explain the lymphopenia in critically ill patients [43,44]. Moreover, recent studies have also correlated lymphopenia with SARS-CoV-2 infection severity [24,25,29,44]. Therefore, the presence of lymphopenia in patients with viral infection, particularly influenza and SARS-CoV-2, could be used as a predictor of disease severity and mandate prompt intervention.

Our results also demonstrated a longer median length of hospital stay for RSV (7 days) compared with influenza and SARS-CoV-2 (5 days). This median length of stay is slightly longer than that reported in similar studies, which found median lengths of stay of 4 days for RSV and 3 days for SARS-CoV-2 [13]. In contrast, our findings are consistent with data obtained at the beginning of the SARS-CoV-2 pandemic, which reported a similar duration of stay for patients infected with influenza and SARS-CoV-2 [25,27]. Moreover, this study is the first study to determine the clinical outcomes and lengths of hospital stay for individuals infected with RSV from the southern region of Saudi Arabia. However, a recent study from the eastern region of Saudi Arabia reported that the average length of hospital stay due to RSV infection was 6.5 days [32]. This slight variation in the duration of stay between these two studies can be attributed to the high altitude of our study region, which necessitates an increased need for oxygen support to maintain saturations of >90% [45].

With respect to PICU admission, it was found that 27.2% of the total number of children required PICU admission, with the median length of stay being 5 days (IQR: 3–8 days). All the children in the PICU required non-invasive respiratory support, and 15.6% required mechanical ventilation. Several clinical factors predicted PICU admission, including age <6 months, RSV infection, CHD, CLD, CNS morbidity, airway anomalies, prematurity and SpO_2_ <90%. In accordance with the present results, previous studies have demonstrated that infants and patients with cardiopulmonary comorbidities have a higher risk of PICU admission compared with older children and those with no comorbidities [46–48]. As these factors have been reported as risk factors in a few similar studies, a well-designed prospective study is needed to clarify the strength of this association and develop a reliable prediction score for healthcare providers to use in caring for these patients, so that they can anticipate complications and prevent poor outcomes. The longer PICU admission was observed in this study for children with RSV infections (35.7%). Interestingly, PICU stay for influenza B patients is also relatively long (24.4%) (as shown in Table 3). We believe this can majorly be attributed to the low number of children diagnosed with influenza B infections as compared to other infections. The severe and critical cases of influenza could be attributed to the pathophysiology associated with influenza viruses’ infections which involve lung cells killing resulting in acute pneumonia [49]. Additionally, the severity of influenza B infection among the paediatric patients in the study area leading to PICU admission could be explained by the lack of routine vaccination policy including influenza type B virus.

These findings cannot be extrapolated to all patients with ARIs in Saudi Arabia due to the following limitations. For instance, it is possible that these results are biased, given the retrospective nature of this study. This study was limited to a single centre with small sample size, which therefore limits the applicability of the findings to other populations and seasons, similar to other observational and hospital-based studies [13]. Generalization of these findings to the whole spectrum of these viral infections is impossible, and further community-based studies are recommended. It is unfortunate that this study did not include other viruses that commonly cause LRIs, such as rhinovirus and human metapneumovirus. Finally, the absence of the whole-genome sequencing of these viruses undoubtedly limits the exact identification of the viral subtypes and their correlations with disease severity. Therefore, genomic analysis studies of these viruses are recommended to compare viral lineages with internationally existing viral lineages [38,39]. Taken together, these findings will be of interest to researchers interested in viral epidemiological trends in the post-SARS-CoV-2 pandemic era.

## Conclusions

This study highlighted the epidemiological and clinical characteristics of four viral pathogens in paediatric patients with acute LRIs in Saudi Arabia in 2022. RSV was identified as the most common pathogen causing acute LRIs among the studied patients. One of the more significant findings to emerge from this study is the seasonal changes in RSV and influenza infections, which mandates further research. To understand the full epidemiological trends and clinical characteristics of these four viruses, additional prospective multicentre studies are needed in order to put forward recommendations for future preventive measures.

## Data Availability

The datasets used in this study are available from the corresponding authors upon request.
